# Quantifying systemic molecular networks affected during high altitude de-acclimatization

**DOI:** 10.1038/s41598-023-40576-w

**Published:** 2023-09-07

**Authors:** Subhojit Paul, Shikha Jain, Anamika Gangwar, Swaraj Mohanty, Nilofar Khan, Yasmin Ahmad

**Affiliations:** Peptide & Proteomics Division, Defence Institute of Physiology & Allied Sciences (DIPAS), Defence R&D Organization (DRDO), Timarpur, New Delhi, 110054 India

**Keywords:** Biological techniques, Computational biology and bioinformatics, Molecular biology

## Abstract

High altitude acclimatization and disease have been the centerpiece of investigations concerning human health at high altitude. Almost all investigations have focused on either understanding and ameliorating high altitude disease or finding better methods of acclimatization/training at high altitude. The aspect of altitude de-induction/de-acclimatization has remained clouded despite the fact that it was documented since the first decade of twentieth century. A few recent studies, particularly in China, have stated unanimously that high altitude de-acclimatization involved multiple observable clinical symptoms ranging from headache to abdominal distention. These symptoms have been collectively referred to as “high altitude de-acclimatization syndrome” (HADAS). However, computational omics and network biology centric investigations concerning HADAS are nascent. In this study, we focus on the quantitative proteo-informatics, especially network biology, of human plasma proteome in individuals who successfully descended from high altitude areas after a stay of 120 days. In brief, the protein list was uploaded into STRING and IPA to compute z-score based cut-offs which were used to analyze the directionality and significance of various identified protein networks as well as the proteins within them. Relevant upstream regulators extracted using computational strategies were also validated. Time-points till the 180th day of de-induction have been investigated to comparatively assess the changes in the plasma proteome and protein pathways of such individuals since the 7th day of arrival at altitude. Our investigation revealed extensive effects of de-induction on lipid metabolism, inflammation and innate immune system as well as coagulation system. This novel study provides a conceptual framework for formulating therapeutic strategies to ease the symptoms of HADAS during de-acclimatization. Such strategies should focus on normalization of lipid metabolism, inflammatory signaling and coagulation systems.

## Introduction

High altitude exploration has been a socio-cultural endeavour, business venture, geo-political statement, sport and spiritual experience to multiple cultures and people since the early phases of human civilisation. It has been well documented that high altitude exposure requires acclimatization, a process that enables hypobaric hypoxia tolerance systemically at the physiological and molecular level. However, equally well-known but not given as much importance, is the process of de-acclimatization/de-induction. In this process, the high-altitude acclimatized individual after a chronic stay at altitude returns to normoxic conditions. It has been reported and discussed without much attention in very early papers (early 1900s) on high altitude biology^1^. In the latter half of 1900s, Balasubramaniam et al.^2,3^ specifically sought to understand clinical effects of altitude induction/acclimatization and deinduction/de-acclimatization on cardiac function but only at the acute time scale. The focus on de-acclimatization was muted with results showing normalized cardiac function. Studies by Savourey et al.^4,5^ focusing on cardio-ventilatory, hematological, hormonal and biochemical changes with greater focus on pre-acclimatization and acclimatization as compared to de-acclimatization also reported minimal changes in these parameters during de-acclimatization. However, a paper by Mori and co-workers revealed that hypoxic effects on cardiac function might remain for at least 1 year after the high altitude exposure^6^. Another study by Scognamiglio et al.^7^ in the 1990s showed a depression of left ventricular performance up to 14 days after high altitude exposure as well as changes in cardiac structural and functional parameters. Neurophysiological complications arising due to de-acclimatization and persisting till 75th day of return to normoxic conditions have also been reported^8^. Possible athletic benefits (hematologic and enzymatic) during de-acclimatization (up to 11 days after return from high-altitude areas) had been reported by Boning et al.^9^. More recent work suggests ethno genetic variability in addition to other more self-evident factors (different conditions of ascent, altitude reached, time scales, etc.) as being responsible for variable responses reported in these older studies to de-acclimatization^10^. An overall fact that emerged from these studies was that de-induction caused sub-clinical but long-term effects in multiple organ systems.

In the more recent decades, high altitude researchers have focused minorly on the health effects of high altitude de-acclimatization syndrome (HADAS). A study under the Altitude Omics umbrella by Ryan and co-authors first described loss of hemoglobin mass just 7 days after return to lower altitude^11^. One of the earlier papers formally describing HADAS and its symptoms was by He et al.^12^ The symptoms were described as “dizziness, fatigability, lethargy, chest distress, edema, amnesia, and other physical signs” which were not “ameliorated after short-term rehabilitation and symptomatic treatment” even after ruling out the possibility of other diseases affecting cardio-respiratory, renal and other organ systems in individuals who recently returned from high altitude areas. A related study also shows that perturbations in the hemoglobin, hematocrit, creatine kinase, RBC mass, PaO_2_, PaCO_2_, SaO_2_ and lactate dehydrogenase levels were correlated with HADAS and HADAS related symptoms were observable till the 100th day of return to low altitude^13^. Further, incidence of HADAS in China were reported to be almost 85% with incidence rates increasing in people who have resided at high altitudes for a relatively longer time, are returning to relatively lower altitudes and performed taxing manual work during their high altitude stay in the above two studies. Previously Zhou et al.^14^ had also performed studies correlating cardiac, respiratory, biochemical and hypoxia signaling parameters to understand the mechanistic basis of de-acclimatization. Continued efforts by He et al.^15^ has led to identification of the inflammatory pathways (mediated by IL-17a, IL-10 and TNF-a) and reoxygenation injury as those responding to therapeutic strategies (Shenqi pollen capsule) against HADAS^16^. In other studies, the role of chemoreceptors on cardiorespiratory function during both acclimatization and de-acclimatization was elucidated by Dempsey et al.^17^ suggesting interlinked nature of HADAS with “sleep high-train low” method of altitude training for athletes and sleep apnea. Another investigation reported sympathetic neural over activity after prolonged high altitude exposure in otherwise healthy humans^18^. A study by Singh and co-authors showed that cholesterol and tri-glyceride levels, testosterone and creatinine clearance rates were perturbed between 30 and 90 days after return from high altitude areas suggesting that almost 3 months are required for biochemical parameters to normalize in lowlanders undergoing de-induction after prolonged exposure (up to 13 months) to high altitude hypoxia^19^. Studies on public health, at least in the armed forces domain, have now begun stating the need to address de-acclimatization as a health and safety event^20^.

As is evident, there is a lack of proteo-bioinformatics studies studying the effects of de-acclimatization (and HADAS) in the intermediate term. This study can provide an in-depth view of molecular aftereffects when the physiological parameters do not show much change. Also, the identification of relevant pathways may help us understand further the effects on energy/lipid metabolism and inflammation pointed out previously and design effective strategies. This study aims to study de-acclimatization using proteo-bioinformatics based methods. We have studied 20 age and gender matched individuals exposed to normoxic baseline (216 m asl); hypoxic altitude (4500 m asl) and normoxic sea-level condition (216 m asl) after the high-altitude exposure. Our findings reveal de-acclimatization to cause multiple molecular perturbations despite absence of any commonly notable physiological symptoms. These can be correlated with the previous studies showing changes in cardio-ventilatory and sympathetic nervous system due to the functional relevance of the pathways highlighted. Our study suggests a focus on finding therapeutic strategies specific to energy/lipid metabolism, inflammation/innate immune function and coagulation pathway to counter the effects of de-induction/de-acclimatization. Also, the ethno-genetic background of various populations should be studied in greater depth in future investigations concerning de-acclimatization or HADAS.

## Materials and methods

### Ethics statement

All study protocols were cross-checked and approved by the institutional ethical committee (Institutional Ethics Committee, Defence Institute of Physiology & Allied Sciences: IEC/DIPAS/DIP-251) in accordance with the Helsinki declaration for human studies. Informed written consent was obtained prior to the study from all participants.

### Experimental design

Twenty male volunteers were matched for age, gender, ethno-genetic background and BMI. Exclusion criteria for our study included: excessive alcohol intake, smoking, cardiovascular complications, respiratory diseases, high blood sugar levels, abnormalities in the hepatic system and any other disease. The individuals were assessed for high altitude illnesses (LLS < 4)^21^ throughout their stay and were found free of them at all stages.

Blood samples were taken at the following time-points: Baseline (BL; normoxic control; 216 m asl); Inducted (hypobaric hypoxia; at altitude of 4500 m asl) at high altitude day 30 (iHAD30) and 120 (iHAD120); and De-inducted after high altitude exposure (normoxia; 216 m asl) at day 30 (dHAD30) and day 180 (dHAD180) before breakfast. Plasma was extracted from the collected blood samples and further analysis using proteomics and immunological techniques was performed. The assimilated data was further analyzed using network biology tools like IPA and STRING (Version 11; https://string-db.org/). At each of the abovementioned time-points, the blood pressure, heart rate and SpO_2_ were also recorded for each individual and presented as Mean ± SD. None of the individuals studied reported any common symptoms associated with any high altitude illnesses and HADAS during the duration of the study.

### Collection of blood plasma and preservation of plasma samples

The volunteers were seated in a comfortable posture while blood was drawn from their antecubital vein. Approximately 30 ml of blood was drawn from each volunteer. The blood was collected in EDTA coated vacutainers. Plasma was extracted by centrifugation at 3000 rpm for 15 min at 4 °C. After collecting the plasma in fresh vials, 1.5 µl PI cocktail was added (P8340; SIGMA ALDRICH; USA) to each vial and cryopreserved.

### DCF-DA assay for oxidative stress

DCF-DA assay, with 2′,7′-Dichlorofluorescein diacetate (DCFHDA; Cat # C400, Life Technologies, USA), was used for assaying the ROS levels in plasma. Plasma samples were diluted 1:50 (v:v) in deionized water. 150 μl plasma (diluted) was then incubated with 10 μM DCFDA (10 μL) at room temperature for 30 min in dark amber tubes. Finally, fluorescence was reported at 487 nm excitation and 530 nm emission wavelengths, respectively using flourimeter (LS45 Luminescence Spectrometer, PerkinElmer, USA). The background normalized fluorescence data was then presented as AU per μl of sample.

### iTRAQ labeled LC–MS/MS

After extraction of plasma, the samples were iTRAQ labeled as previously reported^26^. To briefly recapitulate, 100 µl of plasma was collected per volunteer and pooled according to the appropriate time-point (Baseline; iHAD30; iHAD120; dHAD30 anddHAD180). The concentration of proteins was estimated using Bradford assay. 100 µg of protein per sample was depleted for iTRAQ labeling. As per manufacturer’s protocol, the samples were reduced, alkylated, precipitated and trypsin-digested overnight. The groups were then mass-tagged with iTRAQ labels as follows: Baseline (116); iHAD30 (117); iHAD120 (118); dHAD30 (119) and dHAD180 (121). These samples were then pooled and SCX fractionated. The samples were then desalted, speedvac dried and resuspended in appropriate mobile phase for nano-LC–MS/MS analysis. The chromatography was performed with a Thermo EASY nLC system operating in nano range coupled with Q-Exactive mass spectrometer (Thermo Fisher SCIENTIFIC). A top 15 method utilizing the MS/MS scans of the most abundant 15 ions from a full MS scan of m/z 350-6000 was used to select ions for MS/MS.

### Network biology-based analysis

The protein dataset obtained for differentially expressed proteins with expression values available for all groups was used for further IPA and STRING based analyses. In both, the organism was set as *Homo sapiens*. IPA analysis also involved setting the tissue as plasma. Uniprot IDs and respective gene names of proteins were used as identifiers during the analysis with *p*-value ≤ 0.05 constant throughout the analysis. Ingenuity Pathway Analysis Knowledge Base was the reference database for our analysis of the experimental dataset. IPA based analysis revealed the most significant canonical pathways and their state of up-/down- regulation; upstream proteins; interaction networks between canonical pathways and between proteins as well as cellular locations of the proteins involved in significant networks. IPA analyses was carried out with both the induction groups (iHAD30 and iHAD120) and de-induction groups (dHAD30 and dHAD180) versus Baseline and both de-induction groups versus both the respective induction groups (dHAD30 vs. iHAD30; dHAD180 vs. iHAD120). STRING analysis involved evidence setting; medium confidence; statistical background set as whole genome and utilized MCL clustering (inflation parameter set as 5).

### Statistical techniques used

The physiological data was presented as Mean ± SD. Significance was set at *p* ≤ 0.05. Immunoblotting and biochemical assays were analyzed using 1-way ANOVA. Adjusted *p*-values and *q*-values are listed in the Table1_PN19-18 (Sheet name: Proteins). During IPA analysis, we used the set statistical parameters as provided by the inbuilt algorithm as described by Kramer et al.^22^ consisting of Fisher’s exact *t*-test and Benjamini–Hochberg correction. False discovery rate was set at 0.01 for the MS/MS analysis. GraphPad Prism (ver. 9.5) was used for all statistical analyses.

### Immunoblotting

Plasma samples were diluted 1:10 and separated using 1D gel electrophoresis. Each well had 20 µg of plasma protein. The separated protein bands were then electrophoretically transferred onto PVDF membrane using semi-dry transfer apparatus(GE Healthcare Life Sciences, TE 77 PWR). The PVDF membrane was then blocked using 1% PBS-skim milk or 1% PBS-Tween 20 overnight. The membrane was washed thrice and then primary antibodies (RXR, 33481, SAB; plasminogen, A03674-1, BOSTER; C3, PA1-29715, invitrogen; HBB, FNab03768, Fine Test) were added at appropriate dilutions (1:2000–1:3000). After incubation of 3 h, the primary antibodies were removed and the membrane washed thrice with 1% PBS-Tween 20. Appropriate secondary antibodies were then added at required dilutions ranging from 1:20,000 to 1:30,000 and the membranes incubated for 1.5 h. The secondary antibodies were removed and the membrane was again washed thrice with 1% PBS-Tween 20. Chemiluminescent peroxidase(CPS1120-1KT, SIGMA) was then added and the blots imaged using ChemiDoc XRS + imaging system(BIO-RAD Laboratories, Cat No. 1708265).

## Results

### Physiological parameters during and after high altitude hypoxia exposure

All the volunteers had their age, weight and height recorded at the Baseline (BL). Throughout the duration of the study, at mentioned time-points, their blood pressure, heart rate and oxygen saturation (SpO_2_) were also tabulated (Table 1).

**Table 1:** Physiological parameters of all volunteers at selected time-points.

Groups	BP (in mmHg)			
	Sys	Dia	HR (bpm)	SpO_2_ (%)
BL	117.05 ± 9.4	71.2 ± 9.29	68.37 ± 9.26	97.02 ± 0.78
iHAD30 Days	122.4 ± 10.3	76.7 ± 13.2	84.88 ± 11.9	89.17 ± 2.9
iHAD120 Days	127.48 ± 11.88	84.84 ± 9.21	83.13 ± 12.65	88.63 ± 2.5
dHAD30 Days	121.85 ± 10.09	81.54 ± 9.23	74.09 ± 11.39	98.15 ± 0.37
dHAD180 Days	120.17 ± 11.77	77.27 ± 9.2	73.27 ± 10.76	98.09 ± 0.39

Blood pressure (BP) was measured in mmHg (sys: systolic; dia: diastolic); Heart rate (HR) was measured in beats per minute (bpm) and SpO_2_ was measured as percentage (%). Results plotted as Mean ± SD.

We observed that both heart rate and SpO_2_ levels varied significantly during the high altitude exposure. The heart rate increased and SpO_2_ decreased upon high altitude exposure. However, during de-induction/de-acclimatization phase, we failed to observe any significant changes to either parameter when compared to BL. The changes observed across the normoxic vs hypoxic exposure time-points, irrespective of de-induction, have been previously explained by multiple authors. Thus, we did not observe common physiological parameters to show any abnormalities. This led us to further investigate the absence of any abnormalities in these volunteers who had been exposed to 120 days of hypoxia in the context of their proteomic networks modulating this response.

### Plasma proteome analysis using proteo-informatics pipeline

**Figure 1:**
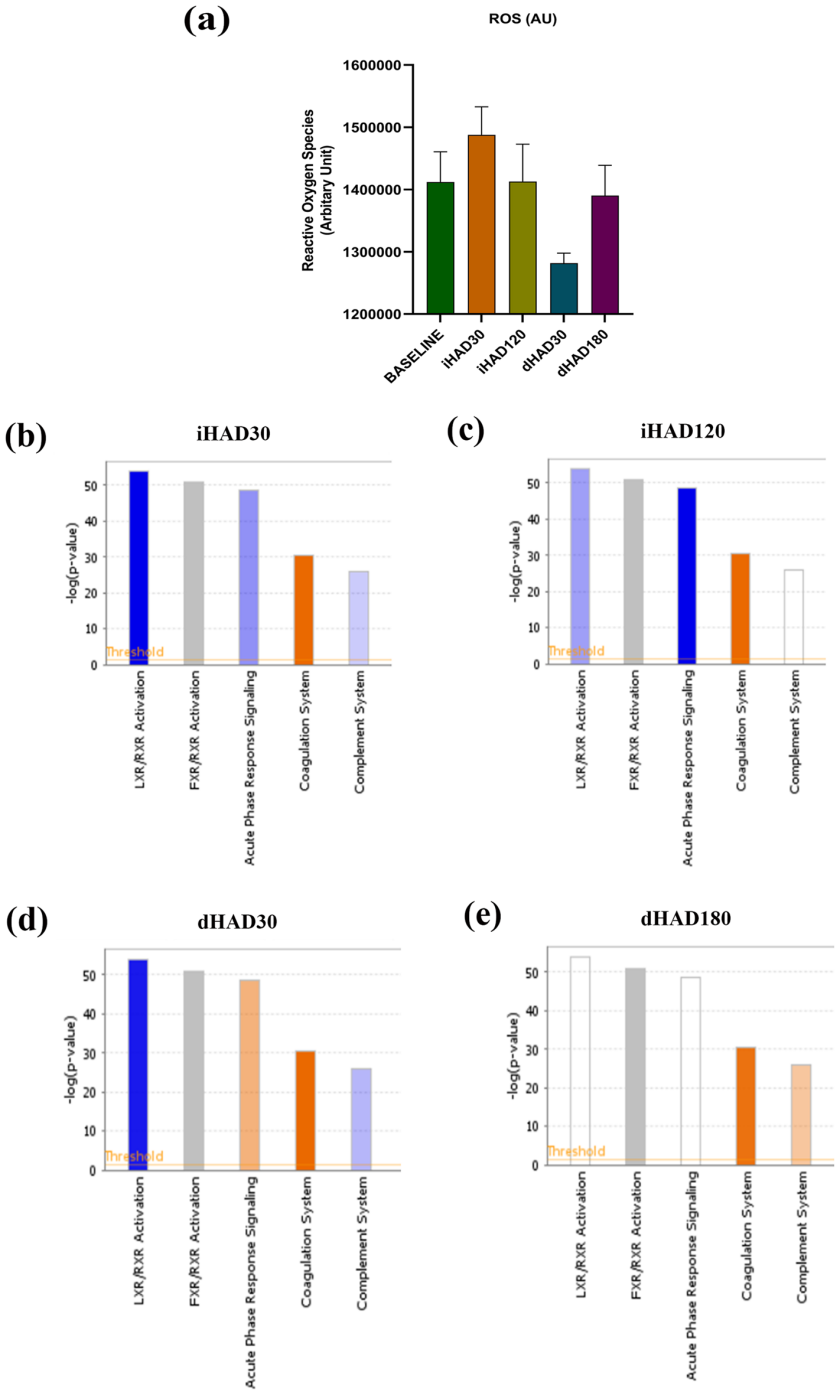
(**a**) ROS levels (mean ± SEM) were measured in Baseline, iHAD30, iHAD120, dHAD30, dHAD180 using DCFH-DA assay. Equal volumes of plasma samples from all volunteers at each time point were pooled and then assayed in duplicates. No statistically significant difference was observed across groups. (**b**–**e**) IPA analysis of LC–MS/MS dataset for iHAD30, iHAD120, dHAD30 and dHAD180 showing top 5 pathways for each group. Trends of the pathways shown as blue (down-regulated); Orange (up-regulated); grey (no trend calculated) and white (neither up- nor down- regulated). Trends of pathways were calculated based on z-scores being > 2 or < -2. All comparisons are w.r.t the BL.

We collected plasma samples from all individuals in this study. At all the time points, the absence of any symptoms of high altitude illnesses and HADAS was confirmed by the volunteers. Before the proteomics-based analysis, we investigated whether ROS fluctuations (a commonly observed phenomenon in hypoxia-induced oxidative stress) were present in the samples using DCF-DA assay. We observed that ROS levels were not significantly altered as compared to BL (Fig. 1a). This indicated that modulation/alteration of various molecular processes, unrelated to ROS modulation, might be occurring upon increasing the partial pressure of atmospheric oxygen as one descends after long-term hypoxic exposure. To further explore this hint, we analyzed the plasma proteome using iTRAQ and LC–MS/MS followed by IPA and STRING analysis. Our initial LC–MS/MS dataset revealed iHAD30 had 8 up-regulated and 2 down-regulated proteins, iHAD120 had 8 up-regulated and 3 down-regulated proteins, dHAD30 had 4 up-regulated and 5 down-regulated and dHAD180 had 22 up-regulated and 6 down-regulated proteins, relative to BL. The fold change cut-off for up-regulation was ≥ 1.5 and down-regulation was ≤ 0.67. Next, we ascertained protein fold change values for the de-induction time points (dHAD30 and 180) relative to their respective induction time points (iHAD30 and 120). In this analysis, we observed that at dHAD30 (vs iHAD30) there were 2 up-regulated and 6 down-regulated proteins. Similarly, at dHAD180 (vs iHAD120), there were 12 up-regulated and 11 down-regulated proteins. Across all the time-points, 246 proteins with differential expression were observed in the LC–MS/MS dataset (Supplementary dataset 1_LC-MS/MS proteins).

### Significant alterations in pathway directionality during de-induction time-points relative to induction time-points

**Figure 2:**
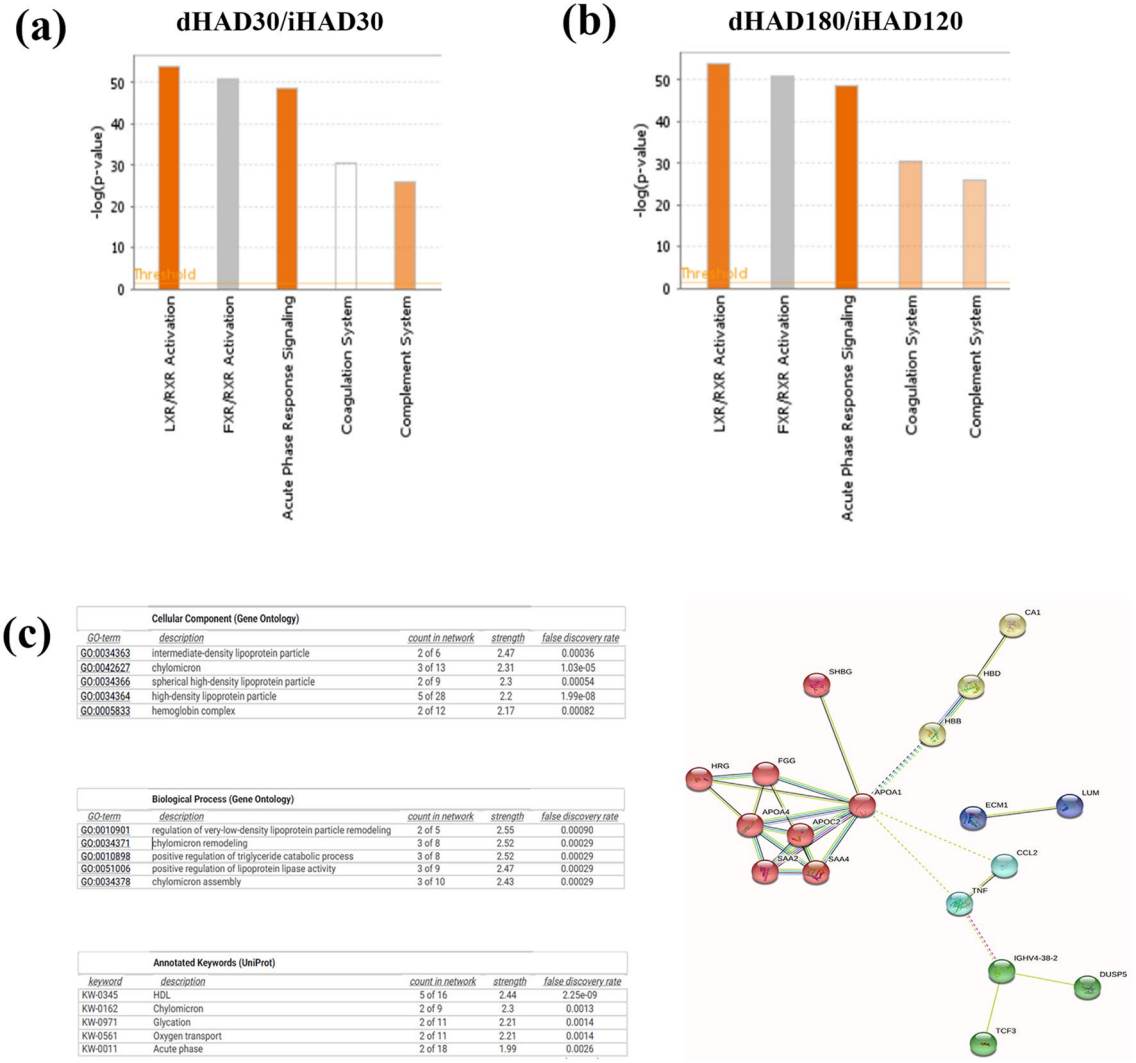
(**a**) IPA analysis of top 5 canonical pathways when comparing dHAD30 vs iHAD30 fold change values (from LC–MS/MS dataset). (**b**) IPA analysis of top 5 canonical pathways when comparing dHAD180 vsiHAD120 fold change values (from LC–MS/MS dataset). (**c**) GO ontology and interaction network analysis(with k-means clustering) using STRING (v 11.5) for dHAD vs iHAD groups.

The blood plasma of the group of volunteers was assessed at normoxic condition (BL; 216 m asl) before their ascent to high altitude; during their high altitude stay (iHAD; 4500 m asl) at days 30 and 120 and finally after their return to normoxic state (dHAD; 216 m asl) at days 30 and 180. Our initial IPA analysis revealed that top 5 Canonical pathways across the iHAD30 (Fig. 1b); iHAD120 (Fig. 1c); dHAD30 (Fig. 1d) and dHAD180 (Fig. 1e) time-points are identical. The directionality of these top 5 pathways during hypoxic exposure at high altitude (iHAD30 and iHAD120) are predictable and linear. In order to better ascertain the directionality of these pathways across induction and de-induction time-points relative to each other, we determined the fold change of dHAD30 proteins relative to iHAD30 protein fold-change values (Fig. 2a; Supplementary dataset 1). We observed that de-induction was indeed causing shifts in directionality of multiple canonical pathways. LXR/RXR Activation, modulating lipid metabolism as well as inflammation, was observed as down-regulated at both dHAD30 and iHAD30. However, relative to iHAD30, LXR/RXR Activation is strongly up-regulated at dHAD30 (Fig. 2a). This suggested increased lipogenesis, lipoprotein synthesis and cholesterol metabolism/transport but it can simultaneously also show increased inflammation. The same is corroborated by the strongly up-regulated acute phase response and complement pathways at dHAD30 (vs iHAD30). This is in line with previous reports of de-induction causing inflammatory signaling^15^. The coagulation system, on the other hand, was neither up-regulated nor down-regulated. This can be traced back to similar scale of up-regulation at both time-points as compared to BL.

Based on our assessment of the relative pathway trends across dHAD30 vs iHAD30, we next determined the pathway directionality of dHAD180 relative to iHAD120 (Fig. 2b). Any stay exceeding 120 days at very high altitude zones (4500 m asl) are considered detrimental to the individuals’ physical and mental health. Also, the objective of this study was to observe the chronic molecular effects of de-induction as clinically observable acute effects of de-induction have been already reported^2,9,13,17^. Similar to our findings in dHAD30 vs iHAD30, we observed that LXR/RXR Activation was up-regulated at dHAD180 (vs iHAD120) as compared to it being neither up- nor down- regulated at dHAD180 relative to BL. Similar trends of up-regulation were observed for the Acute phase response, Coagulation and Complement pathways.

As is very clearly noticeable, there are no significant differences in pathway trends of the top 5 Canonical pathways, except Coagulation system, across the de-induction time-points relative to their respective induction time-points. Coagulation system was down-regulated by dHAD180 (relative to induction time points) but at dHAD30 it was neutral (neither up- nor down- regulated). The same does not hold for comparison of the de-induction time points relative to BL. When compared to BL, at dHAD30, LXR/RXR Activation is strongly down-regulated. But at dHAD180, LXR/RXR Activation pathway becomes neutral. Inversely, the acute phase response pathway, which was up-regulated at dHAD30 became neutral at dHAD180. The Complement system, which was down-regulated at dHAD30, became up-regulated at dHAD180. This suggests a continual perturbation of the lipid metabolic and inflammation pathways even at dHAD180. To further determine which pathways and processes are affected during high altitude de-induction, we assessed the common differentially expressed proteins across the de-induction and induction time points with respect to the entire dataset and their differential expression. A list of 22 differentially expressed proteins were found to be common among the de-induction and induction time points relative to the complete dataset (Supplementary dataset 2_IPA upload sheet). We performed a STRING analysis and observed that the top biological processes were all related to lipid metabolism and transport (Fig. 2c).

### Perturbations in the up-stream regulators and effectors of the LXR/RXR Activation, acute phase response, coagulation and complement pathways

**Figure 3:**
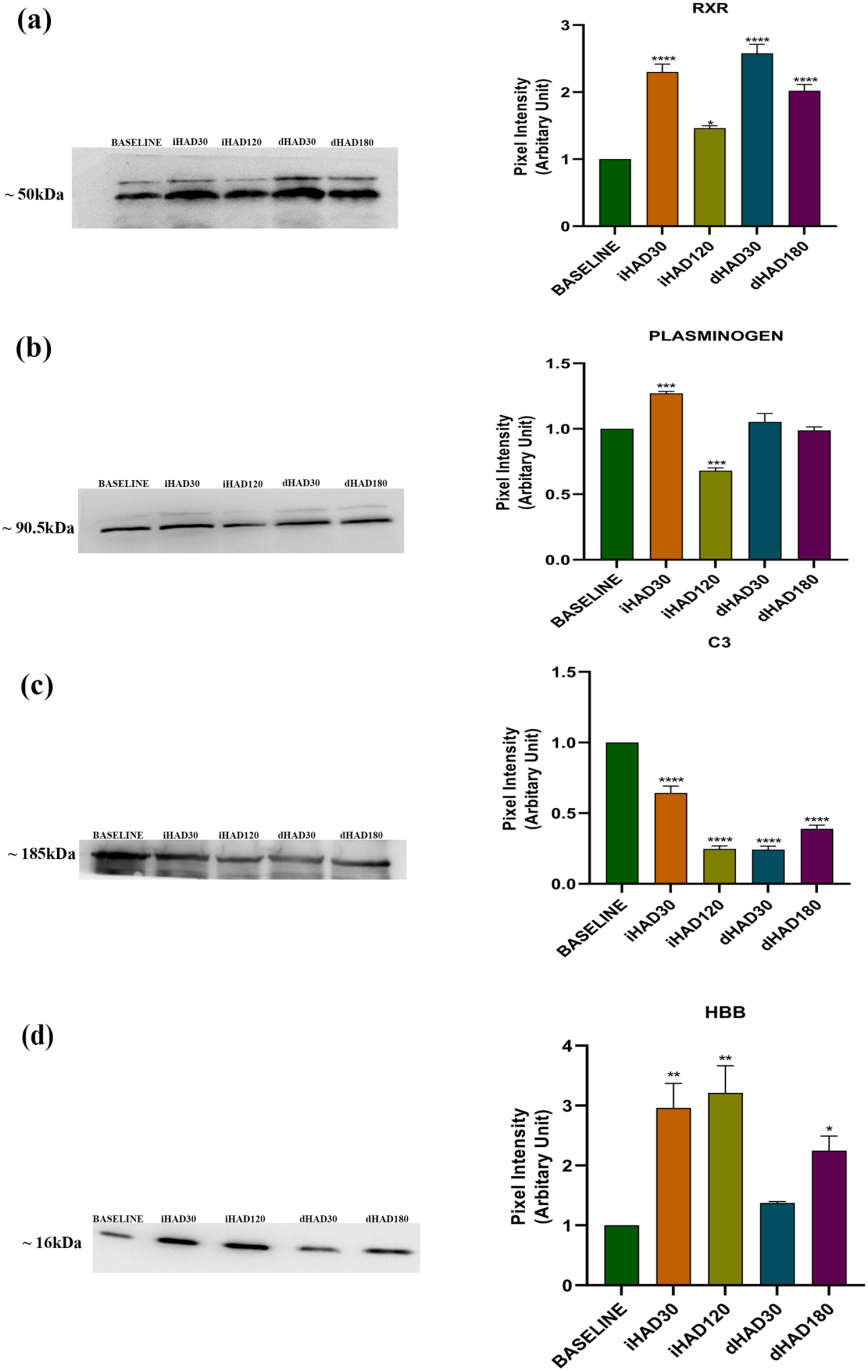
Representative immunoblots and associated bar graphs (Mean ± SEM) for (**a**). Retinoid XReceptor (RXR); (**b**) Plasminogen; (**c**) Complement C3; (**d**) Hemoglobin subunit beta (HBB). *p-value ≤ 0.05; **p-value ≤ 0.01; ***p-value ≤ 0.005; ****p-value ≤ 0.001 All groups were compared to Baseline. Immunoblots were repeated for three biological replicates.

Based on our IPA analysis, we identified the crucial up-stream regulators and effectors in the LXR/RXR Activation, acute phase response, coagulation and complement pathways. These proteins have been listed with their respective pathways (Table 2). We further assessed them using Western blots to determine their expression (Fig. 3). Expression values were correlated with their respective pathways shown earlier (Fig. 1). RXR immunoblots (Fig. 3a) show that increased RXR expression is indicative of down-regulated LXR/RXR Activation pathway while its down-regulation tends to indicate overall neutrality of the pathway, with normoxic conditions favouring a stronger neutral tendency and vice versa (Fig. 1b,c,d,e). Plasminogen (involved in both acute phase response and coagulation pathways) immunoblots (Fig. 3b) show that it is neither highly up-regulated nor down-regulated across the induction and de-induction time-points. This is observed in the constant up-regulation of Coagulation system signaling throughout the same time-points without much change in directionality (Fig. 1b,c,d,e). In addition, we observe that as acute phase response pathway signaling is not dependent on plasminogen trends as it’s only a downstream component. Similarly, for Complement C3 (involved in both complement system and acute phase response pathways) immunoblots (Fig. 3c), we observed that Complement signaling and acute phase response pathways’ directionality is not directly associated with C3 levels as it’s a downstream protein. Based on one of the keywords from STRING analysis being oxygen transport (Fig. 2c), we also assayed hemoglobin subunit beta (HBB) (Fig. 3d), an abundant protein involved in oxygen transport from lungs into tissues. In addition, it’s cleavage product (Spinorphin) is also a selective antagonist of P2RX3 receptor^23^. P2RX3 receptor is involved in platelet aggregation, macrophage activation, pain reception and inflammation^24,25^. HBB trends indicate that high altitude induction triggers its increase, owing to increased oxygen demands in hypoxia. However, its levels remain heightened even after 180 days of de-induction into lower altitude relative to baseline expression. This may suggest improved oxygen transport and pain tolerance after high altitude induction of at least 30 days.

**Table 2:** Proteins selected from the top relevant pathways in IPA analysis for validation using immunoblotting.

Protein	Involved in pathway(s)
RXR	LXR/RXR; FXR/RXR
Plasminogen	Coagulation system
C3	Complement system
HBB	Oxygen transport

*RXR:* retinoid x receptor; C3: complement C3; HBB: hemoglobin subunit beta.

## Discussion

The possibility of descending from altitude (to more hospitable sea-level conditions) inducing any sort of discomfort, let alone any actual health issues, seems bewildering. However, multiple studies from the 1900s till the present decade, confirm HADAS as an actual event during descent from altitude (de-acclimatization). Although the previous studies dealt with de-acclimatization in terms of behavioral and learning tendencies, organ system abnormalities, athletic performance and inflammation/oxidative stress, the proteomic pathways responding to de-acclimatization remain an area still to be explored. In this study, volunteers did not report any symptoms of HADAS or any other extraordinary discomfort. Hence, our study outlines the molecular pathways involved in responding to normal de-acclimatization over a period of 180 days.

We also analyzed ROS levels using the DCF-DA assay. Our results show a slight (not significant) drop at dHAD30 in ROS levels below that observed in the normoxic control group (BL). This is indicative of reductive stress. The levels of antioxidants being too high, leading to accompanying low ROS levels (termed “reductive stress”) have been shown to perturb cell metabolism. A detailed review of reductive stress, wherein the levels of ROS are too low or the levels of reducing antioxidants are too high and its detrimental effects on redox homeostasis is recommended for the readers^26^. Another study in Drosophila stated that low ROS promoted mitochondrial filamentation^27^. It is quite evident that reductive stress is present even after 30 days of deinduction and may have yet unknown effects on mitochondrial signaling. Since the aim of the present study was to explore the effects of de-induction, we intend to pursue low ROS effects and reductive stress outcomes in future studies. The effects of de-induction were further analyzed on the plasma proteome and its relevant pathways using a combination of LC–MS/MS, IPA and STRING^22^. We observed a very clear focus on lipid metabolism, inflammation and coagulation in terms of pathways like LXR/RXR activation, acute phase response signaling and others. In the STRING analysis focused on the common differentially expressed proteins when comparing de-induction with induction and all groups with baseline, we observed that the cellular component was high density and intermediate density lipoprotein particles as well as chylomicrons. Concomitantly, the biological processes associated with these 22 proteins were remodeling, assembly and catabolism of lipoprotein particles (very low density lipoproteins and triglycerides) and chylomicrons.

Our further efforts towards finding the upstream regulators and effectors of these pathways using the IPA upstream regulator function and their subsequent validation at the protein level using immunoblotting led to interesting insights. Juxtaposing the immunoblot trends with the pathway directionality trends, we observed that upstream regulators’ protein expression (e.g. RXR; Fig. 3a) can be used to evaluate overall pathway directionality (LXR/RXR pathway in Fig. 1; inverse trends of immunoblot and pathway directionality). However, the same doesn’t hold for the effector proteins that we assayed, e.g. plasminogen and C3. In case of HBB, an oxygen transporter whose cleavage product Spinorphin selectively represses P2RX3 receptor signaling and modulates diverse processes relevant to acclimatization and de-induction like platelet aggregation, inflammation and macrophage activation, there was no parity between pathway directionality (Acute phase response, Complement and Coagulation pathways; Fig. 1) and protein expression ratios (Fig. 3d). Perhaps, only upstream transcription factors are relevant for assessing pathway directionality. We intend to pursue this as a separate future investigation in hypoxia studies. This study despite its smaller sample size and limited tissue sampling shows that de-induction is indeed causing noticeable molecular perturbations in otherwise healthy and fit individuals. Its implications may help us better understand reductive stress and its effects on the proteome.

## Conclusions

In this study, volunteers were exposed to high altitude conditions for 120 days. Their physiological data and blood plasma samples were collected at sea-level conditions at day 0, high altitude conditions at days 30 and 120 and then at sea level conditions again at days 30 and 180 after the high altitude exposure. We found that even though there were no clinical symptoms of distress reported by the volunteers, the process of de-induction does cause perturbations in the plasma proteome. These perturbations are mostly associated with lipid metabolism, inflammation, reductive stress and coagulation processes. Any therapies for de-induction in the future must strengthen these processes against perturbations during descent from altitude. One of the first processes to investigate should be the reductive stress associated with de-induction followed by the lipid metabolic pathways interconnected to the coagulation, complement and acute phase response cascades.

### Supplementary Information

Supplementary Information 1.

Supplementary Information 2.

Supplementary Information 3.

Supplementary Information 4.

## Data Availability

The data presented in this study are available on request from the corresponding author. The data are not publicly available due to involvement of human subjects and sensitive locations.
