# Supplementary material for: Quantifying systemic molecular networks affected during high altitude de-acclimatization
Source: Sci Rep. 2023 Sep 7;13:14768. doi: 10.1038/s41598-023-40576-w (PMC10484924; doi:10.1038/s41598-023-40576-w)
Supplement: Supplementary file 3 — Supplementary Information 3. [file 41598_2023_40576_MOESM3_ESM.docx]

**Supplementary Files:**

***Section 1: Heart rate & Oxygen saturation Data:***

1. ***Heart Rate (in BPM):***

| **GROUPS** | **Mean** | **Standard Deviation (±)** | **Number of Individuals(n)** |
| --- | --- | --- | --- |
| BASELINE | 68.37 | 9.26 | 20 |
| iHAD30 | 84.88 | 11.9 | 20 |
| iHAD120 | 83.13 | 12.65 | 20 |
| dHAD30 | 74.09 | 11.39 | 20 |
| dHAD180 | 73.27 | 10.76 | 20 |

1. ***Oxygen Saturation[SpO_2_ (in %)]:***

| **GROUPS** | **Mean** | **Standard Deviation (±)** | **Number of Individuals(n)** |
| --- | --- | --- | --- |
| BASELINE | 97.02 | 0.78 | 20 |
| iHAD30 | 89.17 | 2.9 | 20 |
| iHAD120 | 88.63 | 2.5 | 20 |
| dHAD30 | 98.15 | 0.37 | 20 |
| dHAD180 | 98.09 | 0.39 | 20 |


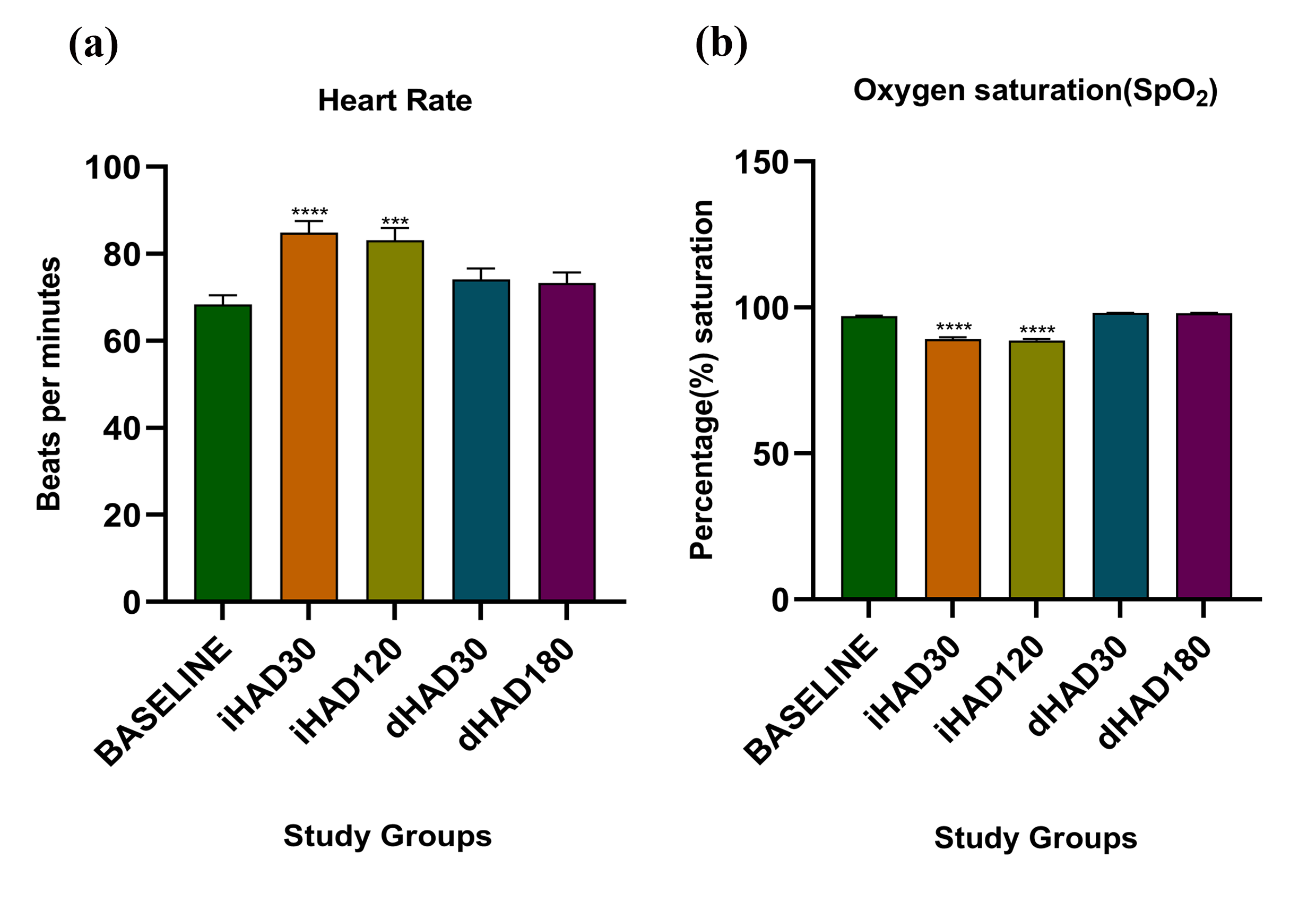


Figures: (a) shows the normalized values plot for Heart rate(in BPM) with adjusted P-values of iHAD30(<0.0001), iHAD120(0.0003), dHAD30(0.3131) and dHAD180(0.4495) w.r.t BASELINE and (b) shows Oxygen saturation(SpO_2_) with adjusted P-values of iHAD30(<0.0001), iHAD120(<0.0001), dHAD30(0.1423) and dHAD180(0.1771) w.r.t BASELINE in statistical analysis of twenty different individuals considered as the subjects for this case study.

***Section 2: Uncropped Western Blot images:***

Please find the supporting files for uncropped images for all the immunoblotting represented in the final figure(Figure 3) of the article used for analysis below for your references.





Figure (a): Shows the uncropped immunoblotting image of RXR (~50kDa) generated from the chemiDoc analysis representing the study groups on the top of the lanes(From Left to Right) as Lane 1: BASELINE, Lane 2: Inducted HAD30(iHAD30), Lane 3: Inducted HAD120(iHAD120), Lane 4: De-inducted HAD30(dHAD30), Lane 5: De-inducted HAD180(iHAD180).

Lane 1 Lane 2 Lane 3 Lane 4 Lane 5





Figure (b): Shows the uncropped immunoblotting image of PLASMINOGEN (~90.5kDa) generated from the chemiDoc analysis representing the study groups on the top of the lanes(From Left to Right) as Lane 1: BASELINE, Lane 2: Inducted HAD30(iHAD30), Lane 3: Inducted HAD120(iHAD120), Lane 4: De-inducted HAD30(dHAD30), Lane 5: De-inducted HAD180(iHAD180).

Lane 1 Lane 2 Lane 3 Lane 4 Lane 5


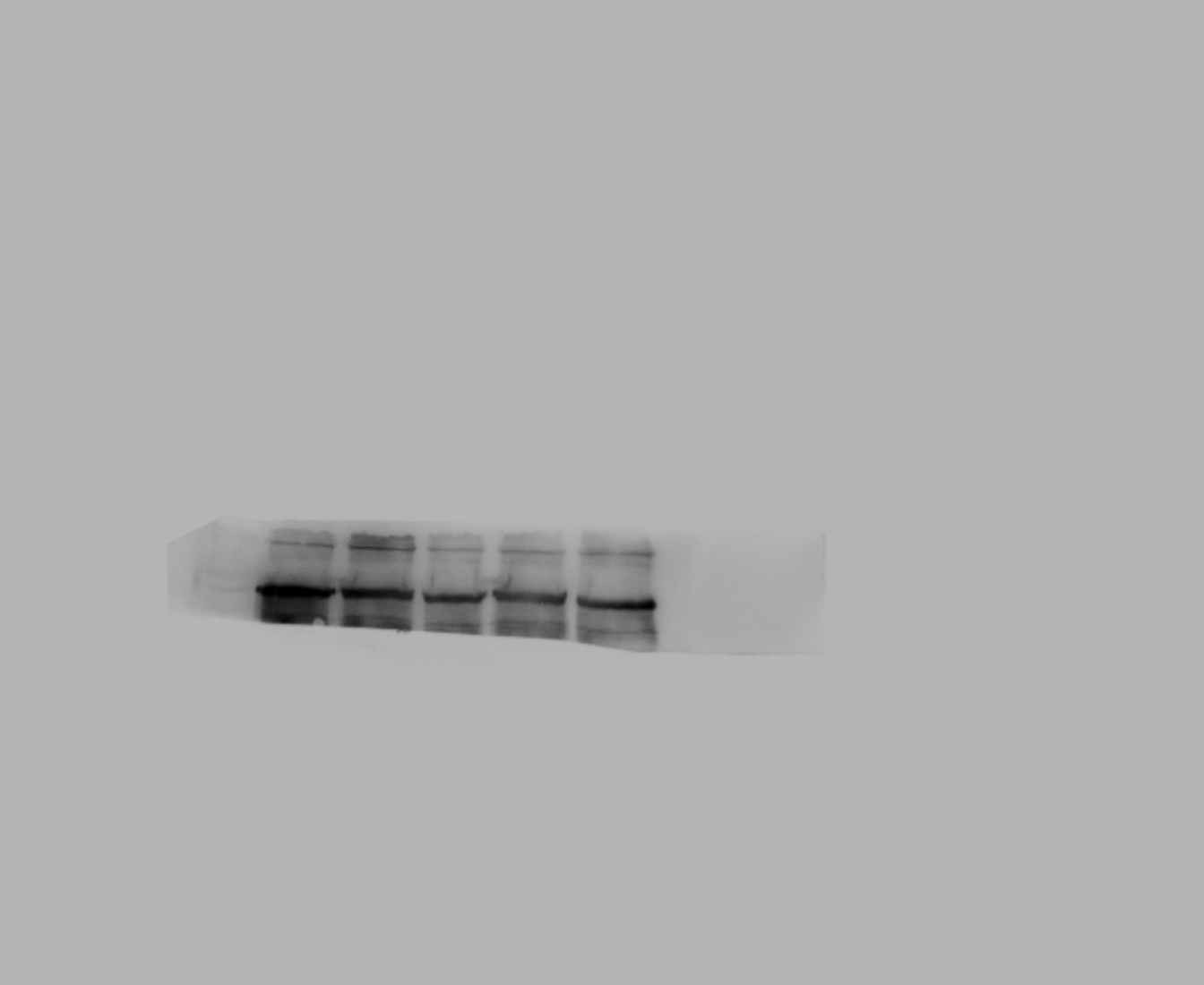


Figure (c): Shows the uncropped immunoblotting image of C3 (~185kDa) generated from the chemiDoc analysis representing the study groups on the top of the lanes(From Left to Right) as Lane 1: BASELINE, Lane 2: Inducted HAD30(iHAD30), Lane 3: Inducted HAD120(iHAD120), Lane 4: De-inducted HAD30(dHAD30), Lane 5: De-inducted HAD180(iHAD180).

Lane 1 Lane 2 Lane 3 Lane 4 Lane 5

Lane 1 Lane 2 Lane 3 Lane 4 Lane 5





Figure (d): Shows the uncropped immunoblotting image of HBB (~16kDa) generated from the chemiDoc analysis representing the study groups on the top of the lanes(From Left to Right) as Lane 1: BASELINE, Lane 2: Inducted HAD30(iHAD30), Lane 3: Inducted HAD120(iHAD120), Lane 4: De-inducted HAD30(dHAD30), Lane 5: De-inducted HAD180(iHAD180).

***Section 3: Housekeeping Gene immunoblot image & Histogram:***


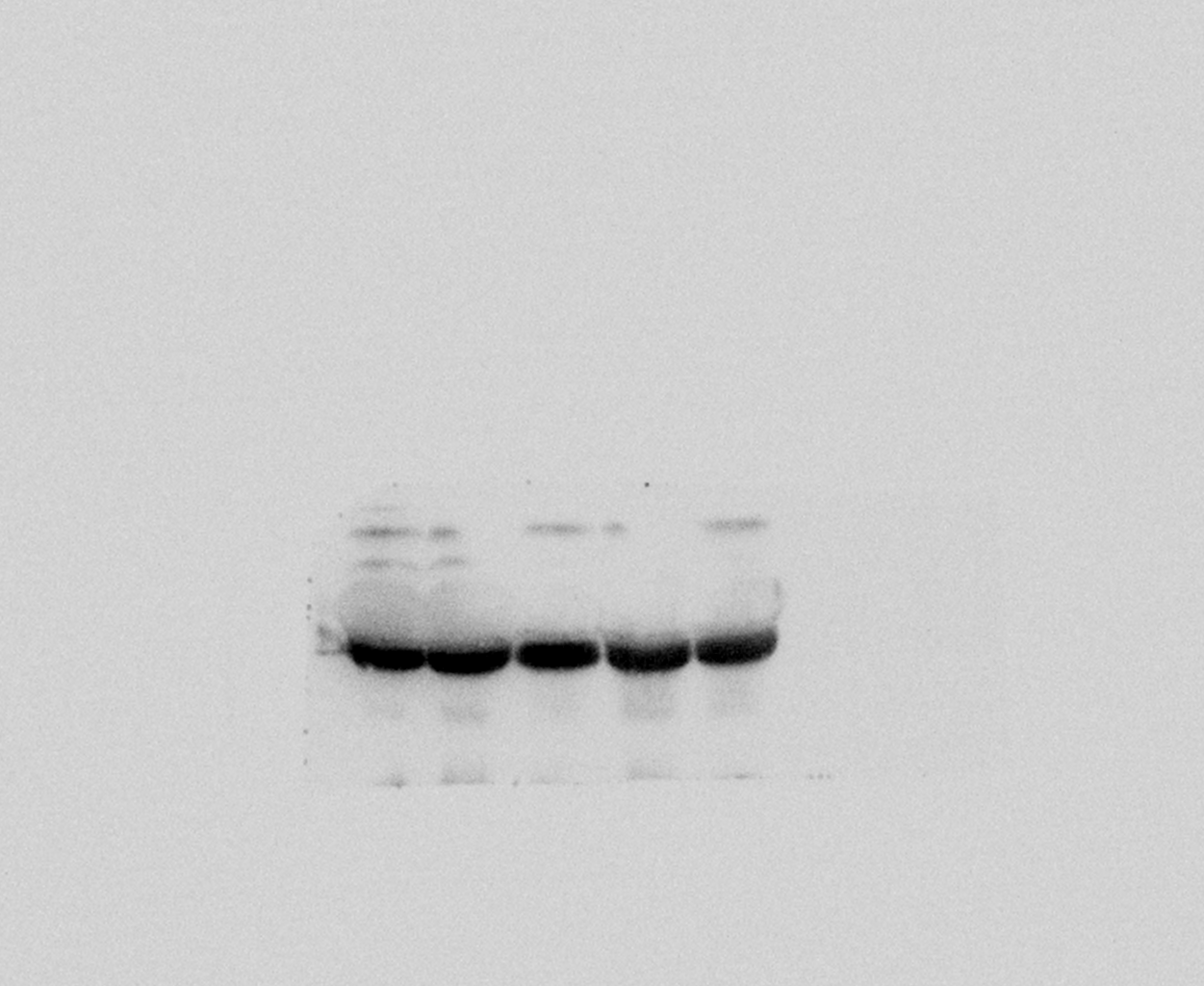


Figure (A): Shows the uncropped immunoblotting image of Tubulin (~16kDa) generated from the chemiDoc analysis representing the study groups on the top of the lanes(From Left to Right) as Lane 1: BASELINE, Lane 2: Inducted HAD30(iHAD30), Lane 3: Inducted HAD120(iHAD120), Lane 4: De-inducted HAD30(dHAD30), Lane 5: De-inducted HAD180(iHAD180).

Lane 1 Lane 2 Lane 3 Lane 4 Lane 5

| **GROUPS** | **Normalized Values** |
| --- | --- |
| BASELINE | 1 |
| iHAD30 | 1.073 |
| iHAD120 | 1.155 |
| dHAD30 | 1.0711 |
| dHAD180 | 0.988 |

Figure (B): Shows the normalized values plot for Tubulin as a housekeeping gene and as a loading control.
